# Risk assessment and suicide by patients with schizophrenia in secondary mental healthcare: a case–control study

**DOI:** 10.1136/bmjopen-2016-011929

**Published:** 2016-09-27

**Authors:** Javier-David Lopez-Morinigo, Rosa Ayesa-Arriola, Beatriz Torres-Romano, Andrea C Fernandes, Hitesh Shetty, Matthew Broadbent, Maria-Encarnacion Dominguez-Ballesteros, Robert Stewart, Anthony S David, Rina Dutta

**Affiliations:** 1 Department of Psychosis Studies, King's College London, Institute of Psychiatry, Psychology and Neuroscience, London, UK; 2 Department of Psychiatry, Marqués de Valdecilla University Hospital, IFIMAV, School of Medicine, University of Cantabria, Santander, Spain; 3 Division of Psychiatry, Virgen de Valme University Hospital, Seville, Spain; 4 Department of Psychological Medicine, King's College London, Institute of Psychiatry, Psychology and Neuroscience, London, UK; 5 Division of Psychiatry, Virgen de la Macarena University Hospital, Seville, Spain

**Keywords:** Suicide, Risk assessment, Schizophrenia, Secondary mental healthcare

## Abstract

**Objectives:**

To investigate the role of risk assessment in predicting suicide in patients with schizophrenia spectrum disorders (SSDs) receiving secondary mental healthcare. We postulated that risk assessment plays a limited role in predicting suicide in these patients.

**Design:**

Retrospective case–control study.

**Setting:**

Anonymised electronic mental health record data from the South London and Maudsley National Health Service (NHS) Foundation Trust (SLaM) (London, UK) linked with national mortality data.

**Participants:**

In 242 227 SLaM service users up to 31 December 2013, 635 suicides were identified. 96 (15.1%) had a SSD diagnosis. Those who died before 1 January 2007 (n=25) were removed from the analyses. Thus, 71 participants with SSD who died from suicide over the study period (cases) were compared with 355 controls.

**Main outcome measure:**

Risk of suicide in relation to risk assessment ratings.

**Results:**

Cases were younger at first contact with services (mean±SD 34.5±12.6 vs 39.2±15.2) and with a higher preponderance of males (OR=2.07, 95% CI 1.18 to 3.65, p=0.01) than controls. Also, suicide occurred within 10 days after last contact with services in half of cases, with the most common suicide methods being hanging (14) and jumping (13). Cases were more likely to have the following ‘risk assessment’ items previously recorded: suicidal history (OR=4.42, 95% CI 2.01 to 9.65, p<0.001), use of violent method (OR=3.37, 95% CI 1.47 to 7.74, p=0.01), suicidal ideation (OR=3.57, 95% CI 1.40 to 9.07, p=0.01) and recent hospital discharge (OR=2.71, 95% CI 1.17 to 6.28, p=0.04). Multiple regression models predicted only 21.5% of the suicide outcome variance.

**Conclusions:**

Predicting suicide in schizophrenia is highly challenging due to the high prevalence of risk factors within this diagnostic group irrespective of outcome, including suicide. Nevertheless, older age at first contact with mental health services and lack of suicidal history and suicidal ideation are useful protective markers indicative of those less likely to end their own lives.

**Strengths and limitations of this study**

- This study used a large clinical database linked to national mortality data to investigate the role of risk assessment in predicting suicide in patients with schizophrenia spectrum disorder (SSD) under secondary mental healthcare over a 5-year period. Those patients with SSD who took their lives over the study period (cases) were compared with a control group drawn from the same database (those participants with SSD who did not die from suicide).
- Suicide risk assessment failed to predict most of suicides, while most of controls were correctly identified.
- Risk assessment appears to be of little relevance for patients with SSD receiving secondary mental healthcare who end their lives.
- Older age at first contact with mental health services and lack of suicidal history and suicidal ideation emerged as useful protective markers indicative of those less likely to end their own lives.
- Risk assessment ratings were unavailable for a number of patients and the risk factors evaluated by this instrument may have changed from the time of assessment to death.

## Introduction

Suicide is a major public health problem with nearly one million deaths across the world per year.^1^ Suicide rates in the UK have remained unchanged over the past 5 years.^2^

Bleuler^3^ ^4^ stated that ‘suicidal drive is the most serious of all schizophrenic symptoms’. The rate of suicide in schizophrenia has been recently reported to be from 2%^5^ to 5%^6^ lifetime risk, which, while lower than the previously quoted estimate of 10%,^7^ ^8^ remains unacceptably high, representing the largest single cause of excess mortality in schizophrenia.^9–11^

Some recognised general suicide risk factors have been replicated in people with schizophrenia such as being male, living alone, depression, hopelessness, previous suicide attempts,^12–14^ number of previous admissions,^14^ self-devaluation, agitation and insomnia.^13^ Interestingly, prior suicidal ideation has been found to be relatively uncommonly reported in patients with psychosis who take later their own lives.^15^ Moreover, schizophrenia has been linked to specific suicide risk factors such as poor treatment compliance.^12^ ^13^ ^16^ Therefore, certain aspects of insight associated with compliance^17^ may prevent patients with psychosis from ending their lives,^18^ and this is supported by a recent 12-month follow-up first-episode study.^19^

With regard to suicide method, patients with schizophrenia have been reported to frequently use violent suicide methods, particularly jumping from a height or in front of a train.^5^ ^20^ ^21^ This has implications for suicide prevention as restricting access to methods has been demonstrated to reduce suicide rates at a population level.^21–24^

Over the past two decades, the UK Department of Health has aimed to reduce suicides at a national level.^25^ In keeping with this, structured clinical risk assessments were strongly recommended by the UK National Institute for Health and Care Excellence (NICE) guidelines in 2004^26^ and widely used. However, recent reviews of the NICE guidelines have voiced concerns about the limited role of risk assessment tools and scales in the clinical management of the patients.^27^ Moreover, a recent meta-analysis showed that risk scales are of little use for predicting repeat self-harm in suicide attempters.^28^ However, the extent to which these instruments can predict suicide risk in patients with schizophrenia spectrum disorders (SSDs) receiving secondary mental healthcare has not been sufficiently examined.^29^ Moreover, concerns have been voiced regarding the role of risk assessment in preventing suicide in patients with schizophrenia.^30^ Also, it remains poorly understood what specific factors evaluated by the risk assessment lead patients with schizophrenia under the care of mental health services to take their own lives.

We aimed to investigate the role of risk assessment in predicting suicide in patients with SSDs receiving secondary mental healthcare. Specifically, risk assessment ratings were compared between patients with SSD who died by suicide (cases) and those attending the same service who did not (controls), while adjusting the analyses for potential confounders, including sociodemographic and clinical variables and ‘service use’-related factors. We postulated that, although previous suicide attempts and suicidal ideation are common risk factors among those who go on to end their lives, risk assessment plays a limited role in predicting suicide in patients with SSD under secondary mental health services due to the high prevalence of the classic risk factors evaluated by these instruments within SSD and suicide being a very rare outcome.

## Methods

### Participants

The sample was derived from the South London and Maudsley (SLaM) Biomedical Research Centre (BRC) Case Register. SLaM is a National Health Service (NHS) Trust which provides secondary mental healthcare to four boroughs in South-East London (UK): Lambeth, Southwark, Lewisham and Croydon. Approximately 1.23 million inhabitants reside in this geographic catchment area, which as a whole was found to be comparable with other populations of London in terms of age, gender, education and socioeconomic status distributions.^31^ ^32^ Full electronic health records have been in use across all SLaM services since 2006, and in 2007–2008 the Clinical Record Interactive Search (CRIS) system was built which renders de-identified copies of records available for research use with appropriate governance structures.^31^ CRIS currently accesses data on over 250 000 patients.^32^ The research ethics approval also covers the pseudonymised linkage between CRIS data and those from the Office for National Statistics (ONS) in January 2014,^33^ which registers all deaths in the UK and the official cause of death, including suicide and the method of suicide according to International Classification of Diseases (ICD)-10 classification.^34^

Those patients who had received SLaM care (ie, had at least one face-to-face contact with a clinical member of staff) before 31 December 2013, had a primary ICD-10 diagnosis of SSD (F2-ICD-10 codes) and who had died by suicide (according to the death certificate) were included as ‘cases’. Those participants with an ‘undetermined cause of death’ (ICD-10 Y) code over the period from 1 January 2007 to 31 December 2013 were considered as cases because in the UK most ‘open verdicts’ are very likely to be suicides.^35^

In addition, five ‘controls’ were selected per ‘case’. Specifically, for each case we selected the next five individuals from the SLaM BRC CRIS who received a primary diagnosis of SSD, had their first face-to-face contact with a SLaM member of the staff after the date of first contact with services of the index case and had not ended their lives by the end of the study period. The controls were unmatched for demographic or clinical variables to allow investigation of between-group differences.

### Measures

#### Risk assessment

‘Full risk assessment’ is a compulsory target across the Trust when ‘high risk’ is determined from a ‘brief risk assessment’, which is mandatory for all active cases. All patients who have been seen by a member of the staff have a ‘brief risk assessment’ documented, which is a narrative record of the patient's risk: (1) to one's self; (2) to others and (3) from others. If the patient is deemed at ‘high’ risk in any of these domains, a ‘full risk assessment’ needs to be completed and updated over time, which consists of a structured assessment taking the form of present/absent tick-boxes enquiring about widely recognised risk factors for three major clusters: suicide, violence and self-neglect. Positive responses can be summed to create total scores, that is, the higher the score the greater the suicide risk.^36^ The most recent full risk assessment was considered for these analyses.

#### Demographic and clinical variables

Demographic/clinical covariates included year of birth, gender, ethnicity, marital status, employment status and ICD-10 diagnosis. In addition, the type of the most recent antipsychotic and/or antidepressant recorded either at the time of death or at the end of the study period (31 December 2013), whichever was sooner, were analysed. Patient legal status under the UK Mental Health Act 1983 (Amended 2007) and being subject to a ‘community treatment order’ (CTO)^37^ were considered.

ICD-10 diagnoses^34^ were reached by consensus by the treating multidisciplinary team, including input from a senior consultant psychiatrist. Specifically, those patients with a diagnosis within the schizophrenia spectrum (ICD-10 codes: F20-F29) were included.

#### Suicide method

Suicide method was ascertained using death certificate^33^ ICD-10 codes^34^ and the following groups were considered: poisoning—X64; hanging—X70; drowning—X71; cutting—X78; jumping (either from high place or in front of a vehicle)—X80, X81; suicide by unspecified means—X84 and undetermined cause of death—Y10-34.

### Statistical analyses

Cases and controls were compared in the following respects: (1) demographic and clinical variables using Student's t-tests, Mann-Whitney U tests and χ^2^ tests, as appropriate; and (2) full risk assessment, where completed, both individual items (χ^2^ test, including ORs and 95% CIs) and total scores (Student's t-test). In addition, receiver operating characteristic (ROC) curves^38^ were plotted to assess the performance, namely sensitivity and specificity, of risk assessment total scores to predict suicide in patients with SSD. Also, the prevalence of suicide in schizophrenia (5%)^6^ was used to estimate the positive and negative predictive values.

The above univariate analyses formed the basis for further multivariable regression models so they were unadjusted. Next, binary logistic regression models were built to investigate inter-relationships between the above variables with regard to suicide. Statistically significant variables from the above univariate analyses were added to a binary logistic regression model. A final regression model was conducted with the independent variables that remained significant. ORs and 95% CIs were calculated. Specifically, the percentage of variance on the dependent variable (ie, suicide) explained by each model (through the Nagelkerke R^2^), the percentage of individuals correctly classified across groups (cases and controls) and the individual contribution of each independent variable to the model (ORs and 95% CIs) were investigated.

A significance level of 5% (two-tailed) was used for all the above analyses, which were performed using SPSS V.21.0 (SPSS, Chicago, Illinois, USA).

## Results

In 242 227 SLaM service users up to 31 December 2013, 635 deaths from suicide were identified. Of these, 96 (15.1%) had a SSD diagnosis. Those who died before 1 January 2007 (n=25) were removed from the analyses, leaving 71 cases who were compared with 355 controls.

Sociodemographic and clinical characteristics of the whole sample are presented in table 1, including comparisons across groups.

**Table 1:** Demographics and clinical characteristics of the sample and between-groups comparisons

	All patients N=426	Cases N=71 (16.7)	Controls N=355 (83.3)		p Value
	Mean±SD	Mean±SD	Mean±SD		
Age at first referral (years)	33.9±21.2	30.3±21.1	34.6±21.2		0.12
Age at first contact (years)	38.4±14.9	34.5±12.6	39.2±15.2		0.01
Age at death (years)	44.9±18.0	38.5±13.2	63.2±17.6*		<0.001
Social deprivation	31.9±11.3	30.2±12.2	32.3±11.1		0.18
	**n (%)**	**n (%)**	**n (%)**	**OR (95% CI)**	
Gender (males)	254 (59.6)	52 (73.2)	202 (56.9)	2.07 (1.18 to 3.65)	0.01
Marital status (unmarried)	387 (90.8)	66 (92.9)	321 (90.4)	1.36 (0.51 to 3.60)	0.65
Unemployed	182 (87.1)	28 (82.3)	154 (88.0)	0.64 (0.24 to 1.72)	0.40
Ethnicity					
White	189 (44.4)	33 (46.5)	156 (43.9)	1.17 (0.66 to 1.85)	0.70
Black	165 (38.7)	25 (35.2)	140 (39.4)	0.83 (0.49 to 1.42)	0.59
South-Asian	24 (5.6)	5 (7.0)	19 (5.3)	1.34 (0.48 to 3.71)	0.57
Others	27 (6.3)	4 (5.6)	23 (6.4)	0.86 (0.29 to 2.57)	0.79
First language English	251 (58.9)	40 (56.3)	211 (59.4)	0.88 (0.53 to 1.47)	0.69

*Cases compared with those controls who died from natural causes (n=25).

Specifically, while age at first referral did not differ across groups, suicidal cases were significantly younger at the time of first contact with services than controls (34.5±12.6 vs 39.2±15.2, t=−2.43, p=0.015). There was a higher male predominance in cases compared with controls (OR=2.07, 95% CI 1.18 to 3.65, p=0.012). However, no group differences in marital status, employment, ethnicity, social deprivation or speaking English as first language emerged from the analyses.

### 

#### Service use-related factors

As detailed in table 2, cases were seen by a member of the staff within a shorter period of time from the first referral than controls (median 20 vs 133 days, respectively), although this difference did not reach significance (p=0.12). Also, cases received significantly shorter duration of care from the Trust teams (median days 1283 vs 2517, p<0.001).

**Table 2:** Service use-related factors of the sample and between-groups comparisons

	Total sample N=426	Cases N=71 (17.7)	Controls N=355 (83.3)		p Value
	Median	Median	Median		
Length from referral to first contact (days)	77	20	133		0.12
Length of service contact (days)	2255	1283	2517		<0.001
Last face-to-face to death (days)	16*	10	63*		0.04
Last hospital discharge to death (days)	233*	161	571*		0.25
	n (%)	n (%)	n (%)	OR (95% CI)	
Antipsychotic					
Oral classic	10 (2.3)	2 (2.8)	8 (2.2)	1.15 (0.24 to 5.58)	0.69
Oral atypical	200 (46.9)	38 (53.5)	162 (45.6)	1.27 (0.65 to 2.49)	0.51
Clozapine	24 (5.6)	2 (2.8)	22 (6.2)	0.39 (0.09 to 1.72)	0.27
Depot	80 (18.8)	12 (16.9)	68 (19.1)	0.75 (0.37 to 1.52)	0.49
Antidepressant	76 (17.8)	16 (22.5)	60 (16.9)	1.43 (0.77 to 2.66)	0.31
SCT	18 (4.2)	3 (4.2)	15 (4.2)		1.00

*Comparing those who completed suicide (n=71) with those who died from natural causes (n=25).SCT, supervised community treatment.

No significant differences were found between cases and controls in type of last antipsychotic medication prescribed or use of antidepressants. Also, the percentage of participants under supervised community treatment did not vary between groups.

#### Suicide method

Hanging (14) and jumping (13) were the most common suicide methods. Twenty-six individuals received an open verdict (undetermined cause of death), as detailed in table 3. There were no suicides by firearms.

**Table 3:** Suicide method (ICD-10 codes)

	N=71
Hanging (X70)	14
Jumping (X81, X81)	13
Poisoning (X60)	4
Drowning (X71)	4
Cutting (X78)	2
Unspecified means (X84)	8
Undetermined (Y10–34)	26

ICD, International Classification of Diseases.

#### Risk assessment

As shown in table 4, 31 cases (43.6%) had previously received at least one full risk assessment completed, and this was significantly lower than in controls (n=214; 60.3%; OR=0.51, 95% CI 0.30 to 0.85, p=0.012). Interestingly, we found a significant time trend in increase of risk assessment completions rates (χ^2^=20.64, p=0.004). In particular, the suicide risk assessment completion rates according to the first year of contact with services were: 34.2% before 2007, 53.0% in 2007, 54.1% in 2008, 51.6% in 2009, 64.3% in 2010, 62.5% in 2011, 57.1% in 2012 and 100% in 2013. Also, those participants with a documented risk assessment had received significantly (p<0.001) longer care (median=2227 days) than those without risk assessment (median=1092 days).

**Table 4:** Full risk assessment: completion rates, individual items and total scores comparison in cases and controls

	Total sample N=245/426 (57.5%)	Cases N=31/71 (43.6%)	Controls N=214/355 (60.3%)		
Individual items	n (%)	n (%)	n (%)	OR (95% CI)	p Value
Suicidal history	69 (28.2)	18 (58.1)	51 (23.8)	4.42 (2.01 to 9.65)	<0.001
Violent method	41 (16.7)	11 (35.5)	30 (14.0)	3.37 (1.47 to 7.74)	0.01
Plan to end life	9 (3.7)	3 (9.7)	6 (2.8)	3.71 (0.88 to 15.69)	0.09
Suicidal ideation	27 (11.0)	8 (25.8)	19 (8.9)	3.57 (1.40 to 9.07)	0.01
Hopelessness	26 (10.6)	6 (19.3)	20 (9.3)	2.33 (0.85 to 6.35)	0.11
Distress	71 (29.0)	11 (35.5)	60 (28.0)	1.41 (0.64 to 3.12)	0.40
No control of life	50 (20.4)	10 (32.2)	40 (18.7)	2.07 (0.90 to 4.74)	0.09
Alcohol/drugs misuse	78 (31.8)	14 (45.1)	64 (29.9)	1.93 (0.90 to 4.15)	0.10
Impulsivity	81 (33.1)	11 (35.5)	70 (32.7)	1.13 (0.51 to 2.49)	0.84
Living alone	95 (38.8)	10 (32.2)	85 (39.7)	0.72 (0.32 to 1.61)	0.55
Poor physical health	71 (29.0)	5 (16.1)	66 (30.8)	0.43 (0.16 to 1.17)	0.14
Significant loss	63 (25.7)	9 (29.0)	54 (25.2)	1.21 (0.53 to 2.79)	0.66
Disengagement	91 (37.1)	14 (45.2)	77 (36.0)	1.46 (0.68 to 3.13)	0.33
Recent discharge from hospital	42 (17.1)	10 (32.2)	32 (15.0)	2.71 (1.17 to 6.28)	0.04
Family history	9 (3.7)	0 (0)	9 (4.2)	NA	0.62
Total score (mean±SD)	3.36±2.36	4.52±2.98	3.19±2.17		0.02

NA, not applicable.

Mean±SD total scores were higher in cases than controls: 4.52±2.98 vs 3.19±2.17 (p=0.02), respectively. Individual items differences are detailed in table 4. The following items were significantly associated with risk of suicide: ‘suicidal history’ (OR=4.42, 95% CI 2.01 to 9.65, p<0.001), ‘previous use of violent method’ (OR=3.37, 95% CI 1.47 to 7.74, p=0.01), ‘suicidal ideation’ (OR=3.57, 95% CI 1.40 to 9.07, p=0.01) and ‘recent discharge from hospital’ (OR=2.71, 95% CI 1.17 to 6.28, p=0.04).

**Figure 1:**
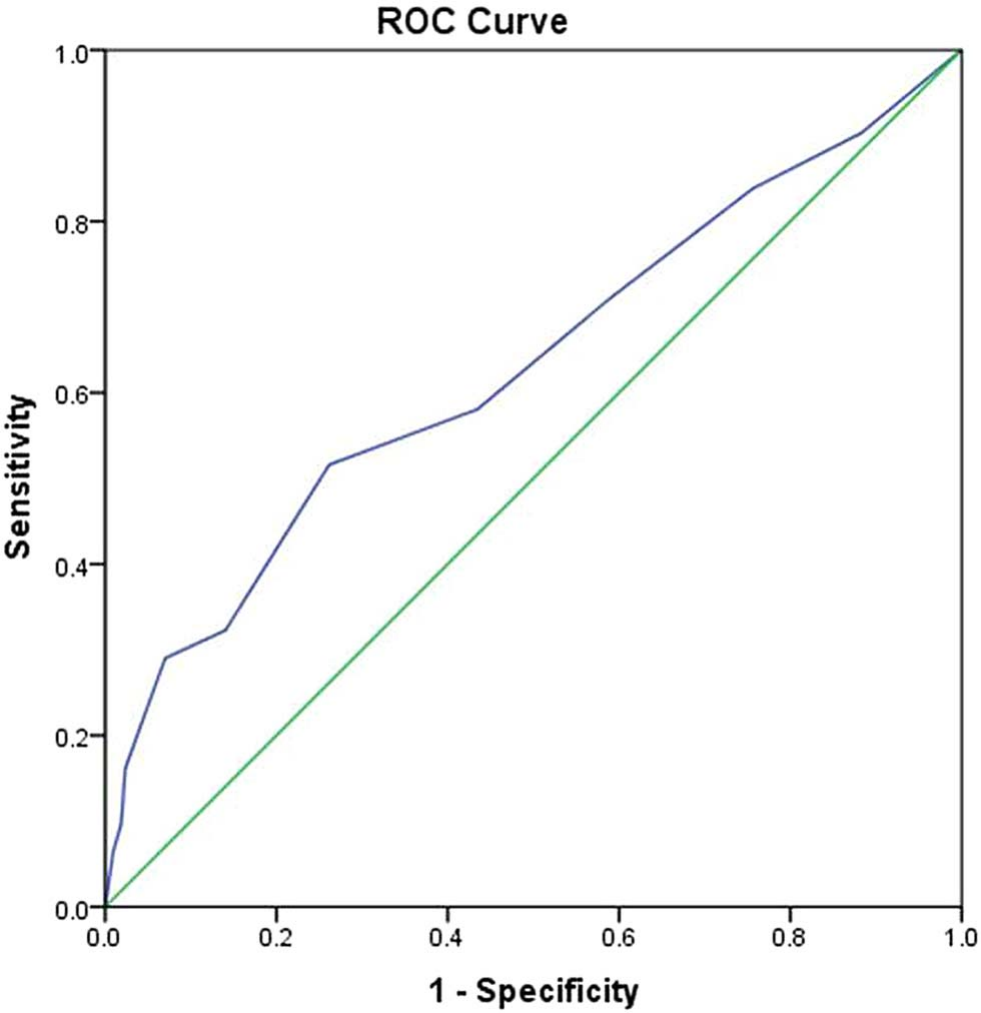
Receiving operating characteristic (ROC) curve for risk assessment total score.

ROC curve analyses for risk assessment total scores found the most optimal cut-off point to be 3–4, with a sensitivity of 0.58 and specificity of 0.57. The area under the curve was 0.63 (95% CI 0.51 to 0.74), which is shown in figure 1 below. If we assume that the prevalence of suicide in these patients is 5%,^6^ the positive predictive value was 0.06, while the negative predictive value was 0.96.

#### Multivariable regression models

Binary logistic regression was performed to examine which variables were associated with suicide. Age at first contact with services, gender and the above four items from the risk assessment (namely, suicidal history, previous use of a violent method, suicidal ideation and recent hospital discharge) were added to the model (see table 5).

**Table 5:** Multivariable regression model: suicide as the dependent variable and all those statistically significant independent variables from the bivariate analyses included

	β	SE	Wald	p Value	OR	95% CI
Age at first contact	−0.064	0.021	8.840	0.003	0.94	0.90 to 0.98
Gender	0.317	0.463	0.470	0.493	1.37	0.55 to 3.41
Suicidal history	1.197	0.558	4.599	0.032	3.31	1.11 to 9.89
Violent method	0.325	0.608	0.285	0.593	1.38	0.42 to 4.55
Suicidal ideation	1.108	0.520	4.539	0.033	3.03	1.09 to 8.39
Recent hospital discharge	0.803	0.481	2.789	0.095	2.23	0.87 to 5.72

However, only age at first presentation (OR=0.94, 95% CI 0.90 to 0.98, p=0.002), suicidal history (OR=4.07, 95% CI 1.80 to 9.18, p=0.001) and suicidal ideation (OR=3.06, 95% CI 1.14 to 8.20, p=0.026) remained significant. The final model (χ^2^=29.771, df=3, p<0.001) explained 21.5% (Nagelkerke R^2^) of the variance on suicide and correctly classified 86.5% of the participants, namely 98.6% of controls and 3.2% of cases (table 6).

**Table 6:** Final regression model: suicide as the dependent variable and only those statistically significant independent variables from the above multivariable regression model

	β	SE	Wald	p Value	OR	95% CI
Age at first contact	−0.064	0.021	9.306	0.002	0.94	0.90 to 0.98
Suicidal history	1.403	0.415	11.420	0.001	4.07	1.80 to 9.18
Suicidal ideation	1.118	0.503	4.939	0.026	3.06	1.14 to 8.20

## Discussion

### Principal findings

In a large clinical case register sourced from electronic mental health records linked to national mortality data, we identified a population of mental health service users with SSD who ended their lives and we investigated risk assessment differences between these individuals (cases) and controls (those who did not take their lives), while adjusting the analyses for potential confounders, including sociodemographic and clinical variables and service use-related factors. In line with our hypotheses, young age at first contact with mental health services, previous suicide attempts and suicidal ideation were associated with suicide. Owing to the rarity of the outcome and limited statistical power, suicide in SSD is not a predictable occurrence, with only 21.5% of the variance explained by the final regression model, yet older age at first contact with mental health services and lack of suicidal history and suicidal ideation are useful protective markers indicative of those less likely to end their own lives.

### Comparison with previous research

#### Risk assessment

Interestingly, suicidal cases were less likely to have a full risk assessment documented, which was somewhat contradictory to previous literature.^29^ However, we found a significant time trend in an increase of suicide risk assessment completion rates over the study years, which suggests that this difference may have been due to the longer care received by controls. Indeed, those patients with a risk assessment completed had been under the Trust teams for a longer time than those participants without risk assessment. Also, a full risk assessment may have been completed due to concerns raised regarding other risks such as violence and/or self-neglect.^36^ Moreover, recording of risk assessment has been reported to be, to some extent, circular. Specifically, some data from risk assessments following self-harm are more likely to be recorded if episodes result in a specialist assessment.^39^

In line with our hypotheses we replicated the role of suicidal history, suicidal ideation, previous use of a lethal method^5^ ^12^ ^16^ ^40^ ^41^ and recent hospital discharge^2^ ^40^ in suicide risk. However, no associations of recorded hopelessness, impulsivity, alcohol/drugs misuse, living alone or significant losses with suicide were found in line with some previous literature on suicide and psychosis.^42^ ^43^ Moreover, most participants with SSD who ended their lives (cases) did not have the factors evaluated by the risk assessment with the exception of ‘suicidal history’. Therefore, suicide in SSD may represent a challenge to the classic suicide model,^44^ particularly regarding the role of hopelessness^44^ ^45^ and impulsivity^44^ ^46^ in suicide, although this finding may be due to the relatively high rates of such factors in SSD per se therefore making it more difficult to ‘pick them out’ against the background. Furthermore, the classic psychosocial factors evaluated by suicide risk assessment instruments were found to be more relevant for patients without SSD who died from suicide.^21^ This finding is consistent with a previous comparison study between suicide attempters with schizophrenia and depression which showed that attempters with schizophrenia had a lower number of life events which were also less influential on the attempt.^47^

Of note, a number of suicides occurred shortly after having been seen by a member of staff, which is in keeping with previous studies showing the relative inability of clinicians to predict and/or prevent imminent suicide risk in individuals with SSD under their care^40^ ^48^ ^49^ despite risk assessment.

In addition, the ROC curves showed that overall risk assessment total scores performed poorly in terms of sensitivity, specificity and positive predictive value, while the test had a very high negative predictive value, which is in full agreement with a recent systematic review of risk assessment scales for predicting repeat self-harms in suicide attempters.^28^ However, at a service level the use of risk assessment tools in NHS-funded hospitals in England was associated with a lower incidence of repeat self-harm at 6 months.^50^

In summary, although suicidal history, suicidal ideation, previous use of a violent suicide method and recent hospital discharge were significantly associated with risk of suicide, the regression models correctly classified most of controls and just a very small proportion of cases, which is in line with our hypotheses and a recent editorial.^30^

#### Sociodemographic differences

Those participants with SSD who died by suicide (cases) had their first contact with mental health services at a younger age than controls, which also remained significant in the multivariable regression models, in line with previous studies showing an increased suicide risk in early psychosis,^4^ although late illness onset reduced suicide risk in an epidemiological study from Taiwan.^50^ Regarding gender, we replicated previous findings from samples of patients with schizophrenia of higher risk of suicide in men,^12^ ^16^ ^40^ ^41^ ^51^ which is also consistent with previous psychological autopsy studies in both psychotic and some, but not all, non-psychotic psychiatric populations.^52^

Interestingly, neither being unemployed nor unmarried was associated with increased suicide risk, consistent with a previous meta-analysis in this diagnostic group.^12^ Also, despite previous literature showing a relationship between ethnicity and suicide in patients with schizophrenia^12^ and in the general population,^53^ we found no evidence of this. This lack of differences in sociodemographic characteristics between cases and controls may have been due to insufficient statistical power. However, it should be noted that the vast majority of patients with psychosis living in South-East London tend to be single, unemployed, socially deprived and of black ethnicities.^54^ ^55^ Moreover, a previous population-level community-based survey conducted in South-East London found poor mental health, low socioeconomic status and being unmarried to be associated with a history of previous suicide attempts, while black African ethnicity was protective.^56^ Although we failed to replicate these findings in our sample of patients with schizophrenia under secondary mental healthcare who took their lives, our previous comparison study of participants under secondary mental healthcare who died by suicide had reported significantly higher levels of social deprivation in patients with SSD than in those with non-SSD diagnoses.^21^ Therefore, there are grounds to consider that low socioeconomic status, which is strongly linked with psychosis in our catchment area,^54^ ^55^ may increase suicide risk in these patients.

#### Service use-related factors

Of note, cases tended to have a shorter interval than controls between the referral and first contact with services. This finding is in full agreement with a previous first-episode psychosis study which showed that suicidal behaviour preceding first contact with services may shorten the duration of untreated psychosis via leading the patient to receive psychiatric attention earlier.^57^ Also, the duration of care was significantly shorter in those who died from suicide (cases), which is likely to have been an artefact due to the survival effect among controls. We replicated the high risk of suicide in patients with schizophrenia during the immediate period after hospitalisation.^2^ ^16^ ^40^ ^41^ ^48^ A third of suicides occurred in the 6-month period after being discharged from a psychiatric ward, which is of particular concern in patients with a first-episode psychosis.^58^ Hence, close monitoring over that period of time should be strongly recommended.^2^ ^14^

Despite the well-known antisuicidal properties of clozapine^59^ ^60^ and some previous recommendations promoting the use of antidepressants^41^ ^61^ and depot antipsychotics^41^ for suicide prevention in schizophrenia, we found no significant differences between cases and controls in the use of these pharmacological treatments. However, our negative results were purely observational and consistent with previous case–control studies in schizophrenia,^16^ ^51^ which all may have had insufficient statistical power.

Receiving community mental healthcare under restriction in accordance with the UK Mental Health Act 1983 (Amended 2007),^37^ which is known as a CTO, showed no association with suicide. These findings seem to be consistent with the UK National Confidential Inquiry into Suicide and Homicide report,^2^ according to which there were 42 suicides in patients subject to a CTO between 2009 and 2013 in England. The suicide rate in CTO patients (2.0 per 1000 CTOs in 2009–2012) was higher than the suicide rate for all patients (0.09/1000-year), and this is not unexpected, since one criterion for selection of patients for CTO is risk on discharge.

#### Suicide method

Regarding suicide method, we replicated that hanging and jumping (from a height or in front of a vehicle) were the most common suicide methods^5^ ^16^ ^20^ ^24^ ^40^ ^42^ ^62^ in SSD. However, patients with SSD were reported to kill themselves by taking overdoses in Finland.^48^ Of note, no suicides by firearms were identified in our study, which is in line with previous reports from Europe,^4^ ^15^ reflecting the restrictions to firearms compared with the USA.

Limiting availability of lethal methods has been demonstrated to reduce suicide rates at a population level.^22^ ^24^ Also, restricting access to suicide hotspots such as heights through safety barriers^23^ and railway lines by installing platform edge doors^63^ has been reported to reduce overall suicide rates at such places.^64^ Hence, installation of physical barriers on bridges, tall buildings and railway stations, particularly near psychiatric hospitals given our replication finding concerning the increased suicide risk after hospitalisation^2^ ^16^ ^40^ ^41^ ^48^ ^58^ may prevent patients with SSD from suicide.^21^

### Strengths and weaknesses

This study focused on the rare outcome of suicide. By using a large case register linked to national mortality data, all those patients with a diagnosis of SSD who were receiving secondary mental healthcare in our catchment area and died by suicide over 2007–2013 were included in the study with the only exception of those who ended their lives outside the UK. Most patients were followed up over a prolonged period (median=6.17 years). As only a tiny proportion of patients living in South-East London receive private mental healthcare, our sample is likely to be representative. In addition to risk assessment, a wide range of demographic and clinical variables, including service use-related factors, were analysed.

However, our results should be considered in the light of several limitations. First, the sample was formed of secondary mental health services users living in South-East London, an inner urban area, and results may not generalise to people receiving mental health input from primary care or those in rural areas. Second, risk assessment ratings were unavailable for a number of patients. Also, we can speculate that those patients who had a risk assessment completed were deemed ‘at high risk’ by their clinical teams. Hence, the likelihood that these measures and findings relating to speed of assessment reflect the clinical response to perceived suicidal risk rather than potential predictors. In addition, although just the last risk assessment was considered, risk factors evaluated by this instrument may have changed from that point to death. Also, it should be noted that a wide range of variables have been taken over a prolonged period of time, which also varies across the study patients, who ranged from having one single assessment to several years under secondary mental healthcare, thus reflecting the real-world nature of our data. In addition, other non-tested variables such as premorbid personality and premorbid adjustment may have contributed to suicide in our sample. Finally, our control recruitment method explained above did not include an algorithm for randomisation of cases and controls.

## Conclusions

Our findings suggest that the classic suicide risk assessment and prevention approach in secondary mental healthcare appears to have little relevance for patients with SSD where many traditional risk factors are prevalent across the board. Of note, a high number of these tragic events occurred shortly after a contact with a mental health professional, hanging and jumping being the most common suicide methods. Older age at first contact with mental health services and lack of suicidal history and suicidal ideation are helpful markers indicative of those less likely to end their own lives.

Hence, a successful approach for suicide prevention is likely to require a combination of both population-level strategies such as restricting access to lethal means^22^ ^24^ and measures focused on high-risk groups such as patients with SSD,^43^ including compliance and engagement improving interventions.^17–19^
