# Correction

**DOI:** 10.1136/bmjopen-2016-011929corr1

**Published:** 2016-11-29

Lopez-Morinigo J-D, Ayesa-Arriola R, Torres- Romano B, *et al.* Risk assessment and suicide by patients with schizophrenia in secondary mental healthcare: a case–control study. *BMJ Open* 2016;6:e011929. doi:10.1136/bmjopen-2016-011929

Dr Rosa Ayesa Arriola's affiliation was published incorrectly. It should be as follows:

Department of Psychiatry. Marqués de Valdecilla University Hospital, IDIVAL, School of Medicine, University of Cantabria, Santander, Spain. Centro Investigación Biomédica en Red de Salud Mental (CIBERSAM). Madrid, Spain

